# Public Knowledge and Perception of Anesthesia, Anesthesiologists’ Expertise, and Their Role Among Saudi Citizens Residing in Saudi Arabia

**DOI:** 10.7759/cureus.44265

**Published:** 2023-08-28

**Authors:** Nehal G Almutairi, Mohammad H Alorainy, Abdulrahman M Aldayel, Haifa A Alotaibi, Sultan A Alkathami, Waleed A Alghamdi

**Affiliations:** 1 Medicine, College of Medicine, Imam Mohammad Ibn Saud Islamic University, Riyadh, SAU; 2 Anesthesiology, College of Medicine, Imam Mohammad Ibn Saud Islamic University, Riyadh, SAU

**Keywords:** saudi population, anesthesia perception, patient trust in anesthesiologists, patient fear of anesthesia, anesthesiologists clinical role, anesthesia knowledge

## Abstract

Background: Despite improvements in anesthesia practice, there is still a lack of public awareness of the field, the range of an anesthesiologist's duties, and the crucial role they play in the healthcare delivery system. Thus, this study aimed to assess Saudi citizens' perceptions of anesthesiologists' training, expertise, role, and responsibilities, as well as their knowledge and concerns about anesthesia.

Method: A cross-sectional study was conducted between December 2022 and April 2023, with a 42-question survey administered to 406 adult Saudi citizens of both genders residing in Saudi Arabia, excluding healthcare students and employees.

Results: Most participants were female (82.8%), aged over 40 (67.6%), held a bachelor's degree (74.6%), and reported very good health (38.7%). A majority (67.2%) had at least undergone one or more surgeries. Knowledge scores averaged 8.14 ± 2.35/14, distributed as 20% poor, 67.7% moderate, and 12.3% good. Perception scores averaged 3.25 ± 1.59/7, with 55.2% poor, 38.2% moderate, and 6.7% good. A significant positive correlation between perception and knowledge scores was found. Higher perception scores were associated with having a chronic medical condition, while higher knowledge scores were associated with being female and having undergone more surgeries. Anesthesiologists were recognized as specially trained doctors by 79.8% of participants, and 63.8% trusted physicians for care. However, 22.4% refused care. Notably, the most common anesthesia concern was fear of dying during anesthesia (very concerned: 26.6%).

Conclusion: This study reveals knowledge gaps and misconceptions about an anesthesiologist's role and responsibilities, highlighting the need for public education to address concerns, improve patient satisfaction, and inform future research.

## Introduction

The perception of anesthesia as a 'behind-the-scenes' expertise has persisted over time. Despite improvements in anesthesia practice, there is still a lack of public awareness of the field, the range of an anesthetist's duties, and the crucial role anesthesiologists play in the healthcare delivery system. Numerous earlier studies conducted in the United States, Europe, and Australia have demonstrated that there is little public understanding of anesthesiologists' training, experience, role, and function both within and outside the operating room [1-3]. Another study conducted in Saudi Arabia reflected the ignorance of the public about the function of anesthesiologists, a lack of perception regarding anesthetic procedures during surgery, and the role of the anesthesiologist in monitoring resuscitation and postoperative analgesia [4]. A more recent study in Saudi Arabia revealed a relative lack of information about the role of the anesthesiologist both intraoperatively and postoperatively [5]. These elements could increase preoperative anxiety and lower post-anesthesia patient satisfaction. In addition, another study in Saudi Arabia revealed that all patients (100%) after receiving an explanation of anesthesia were found to be afraid of it; hence, it was suggested that periodic surveys every five to 10 years may be helpful to gather feedback from the public on their awareness about anesthesiology and anesthesiologists to further enhance it [6], which in return added more value to this current study and its importance.

Surgery and anesthesia are daily procedures for anesthesiologists, but they are also quite worrisome for the patient and their family. There may be adverse effects from this worry that are harmful. Due to the specifics of anesthesia practice, anesthesiologists must overcome communication barriers. Typically, anesthesiologists don't spend much time with conscious patients. Assessments of communication skills should be a crucial component of the residency training program, just like in North America and Britain, because excellent physician-patient communication (verbal and nonverbal) influences factors such as patient satisfaction, patient compliance, and medical results [7]. Without much information about the patient's personality, the anesthesiologist has to provide the patient with a very personalized and intimate level of care. During anesthesia, the patient is kept under control and monitored throughout with the help of procedures that lessen consciousness, induce amnesia, and restrict autonomous movements. 

It is important for anesthesiologists to establish relationships with patients both preoperatively and throughout the surgery. Pre-anesthesia evaluation, periprocedural management, and post-anesthesia care are the three separate phases of anesthesia care and the anesthesiologist-patient relationship. Every stage has different communication difficulties for the anesthesiologists. There are opportunities to give accurate information on anesthesia and anesthesiologists, which can improve results [8]. Thus, this study aimed to assess Saudi citizens' perceptions of anesthesiologist training, expertise, role, and responsibilities, as well as their knowledge and concerns about anesthesia.

## Materials and methods

Study design and setting

An analytical cross-sectional study was conducted between December 2022 and April 2023 using an online survey distributed to Saudi citizens aged 18 years and older living in various regions of Saudi Arabia, irrespective of their gender.

Sampling technique and participants

The study sample consisted of 406 participants from the general public in Saudi Arabia, aged 18 years and older, encompassing both genders. The selection process involved the implementation of specific inclusion and exclusion criteria. The inclusion criteria were defined to include individuals who met the age requirement and were citizens of Saudi Arabia. Non-Saudi participants, healthcare professionals, and individuals younger than 18 years old were excluded from the study. 

Study measurements

The questionnaire utilized in this study was adapted from a previously published study designed by a senior anesthesiologist with input from other anesthesiologists. The questionnaire was modified to suit the objectives and population of the current study. It included demographic data such as gender, age, education, chronic medical conditions, self-reported health, and previous surgeries. The questionnaire assessed the perception of anesthesiologists’ education, expertise, role, and responsibilities (see Appendix A) [1]. It included seven questions, with one correct answer identified according to the training and education in Saudi Arabia by a senior consultant. The questionnaire also evaluated trust in physicians and anesthesiologists, using four questions taken from the *Trust in Physician Scale*, which had been validated in previous studies (see Appendix B) [9 -11]. Fears or concerns about anesthesia were measured using a 10-Likert scale. These questions were related to concerns about the major and minor risks of anesthesia (see Appendix C). Knowledge related to anesthesia was assessed using a set of 14 true/false/I don’t know questions, which covered topics related to the expertise of anesthesiologists, general facts about anesthesia, important preoperative information and instruction, potential side effects or risks, and the overall safety of anesthesia (see Appendix D). [1]

The perception and knowledge of anesthesia were assessed using a 21-item questionnaire that was reviewed by an anesthesia consultant. Of these questions, 7 items were related to perception, and 14 items were related to knowledge. Each question was coded with 1 for a correct answer and 0 for an incorrect answer. The total score for each domain was calculated by adding all questions related to that domain. A higher score indicated a higher level of perception or knowledge about anesthesia. Cutoff points of 50% and 75% were used to categorize the levels of perception or knowledge. Scores less than 50% were considered poor, scores between 50% and 75% were considered moderate, and scores above 75% were considered good.

Statistical analysis

Descriptive statistics were used to define the proportion of responses for each respondent. Values were computed and reported as numbers and percentages for all categorical variables, while the mean and standard deviation were used to report all continuous variables. The differences in the perception and knowledge scores in relation to participants’ socio-demographic characteristics have been analyzed using the Mann-Whitney Z-test. Statistical collinearity was measured using the Shapiro-Wilk test as well as the Kolmogorov-Smirnov test. The Spearman correlation coefficient was used to determine the correlation between perception and knowledge scores. Statistical significance was identified at p<0.05. The data analyses were performed using SPSS Statistics version 26 (IBM Corp., Armonk, NY, USA).

Ethical consideration

The conduct and data collection of this study received ethical approval from the Institutional Review Board (IRB) committee of Imam Muhammad Ibn Saud Islamic University (approval no. 425/2023). 

## Results

The study included a total of 456 participants, out of which 406 were eligible for analysis. However, 50 participants who were healthcare students or practitioners were excluded from the analysis due to their professional backgrounds. The socio-demographic data of the included participants are provided in Table 1.

**Table 1:** Sociodemographic characteristics of the participants (n=406)

Study Data	N (%)
Age group	
18 - 29 years	65 (16.0%)
30 - 39 years	67 (16.5%)
40 - 49 years	105 (25.9%)
50 - 59 years	142 (35.0%)
≥60 years	27 (06.7%)
Gender	
Male	70 (17.2%)
Female	336 (82.8%)
Educational level	
Uneducated	02 (0.50%)
Primary school	01 (0.20%)
Middle school	04 (01.0%)
High school	42 (10.3%)
Bachelor’s degree	303 (74.6%)
Postgraduate	54 (13.3%)
Region of residence	
Central Region	345 (85.0%)
Eastern Region	13 (03.2%)
Southern Region	10 (02.5%)
Western Region	38 (09.4%)
Chronic medical condition (e.g. HTN, DM)	
Yes	94 (23.2%)
No	312 (76.8%)
Self-assessment of health status	
Fair	46 (11.3%)
Good	113 (27.8%)
Very good	157 (38.7%)
Excellent	90 (22.2%)
Previous surgeries	
None	133 (32.8%)
One surgery	112 (27.6%)
Two surgeries	76 (18.7%)
Three or more surgeries	85 (20.9%)

Four hundred and six participants completed the survey. Table 1 presents the socio-demographic characteristics of the participants. Around 35% were aged between 50 and 59 years, with females being dominant (82.8%). Nearly three-quarters (74.6%) had bachelor’s degrees and mostly lived in the Central Region (85%). The proportion of participants with chronic diseases was 23.2%. Perceived health status was very good among 38.7%. In addition, 20.9% underwent three or more surgeries.

Perception of anesthesiologist education, expertise, role, responsibilities, education and training

Table 2 shows that 83.5%, 43.1%, and 30.3% were aware that the anesthesiologist was responsible for putting the patient to sleep, waking him up, and monitoring vital signs before, after, and throughout the surgery, respectively. Around 47.5% and 75.6% knew that the number of medical school years required for anesthesiologists and surgeons was five years or more. However, 26.1% and 18.5% were aware that the number of residency training years required for anesthesiologists and surgeons was four years or less. Based on the above statement, the overall mean perception score was 3.25 (SD 1.59), with poor, moderate, and good perception levels found in 55.2%, 38.2%, and 6.7%, respectively.

**Table 2:** Assessment of perceptions on anesthesiologist education, expertise, role, responsibilities, education, and training (n=406) * Indicates correct answer

Assessment questions/statements & answers	N (%)
Who puts you to sleep before surgery?	
Surgeon	30 (07.4%)
Anesthesiologist *	339 (83.5%)
Nurse	17 (04.2%)
I don’t know	20 (04.9%)
Who is responsible for waking you up after surgery?	
Surgeon	18 (04.4%)
Anesthesiologist *	175 (43.1%)
Nurse	156 (38.4%)
I don’t know	57 (14.0%)
Who is responsible for monitoring your vital signs throughout surgery?	
Surgeon	32 (07.9%)
Anesthesiologist *	123 (30.3%)
Nurse	183 (45.1%)
I don’t know	68 (16.7%)
Statement about education and training	
The number of medical school years required for an anesthesiologist	
4 or less	34 (08.4%)
5 or more *	193 (47.5%)
I don’t know	179 (44.1%)
The number of medical school years required for surgeons	
4 or less	06 (01.5%)
5 or more *	307 (75.6%)
I don’t know	93 (22.9%)
The number of residency training years required for an anesthesiologist	
4 or less *	106 (26.1%)
5 or more	81 (20.0%)
I don’t know	219 (53.9%)
The number of residency training years required for a surgeon	
4 or less *	75 (18.5%)
5 or more	138 (34.0%)
I don’t know	193 (47.5%)
Total perception score (mean ± SD)	3.25 ± 1.59
Level of perception	
Poor	224 (55.2%)
Moderate	155 (38.2%)
Good	27 (06.7%)

Trust among physicians and anesthesiologists

Table 3 reveals that 63.8% put their trust in their doctors in relation to medical care. However, 22.4% had rejected receiving medical care due to a lack of faith in physicians. The majority of our respondents (51%) do not believe that medical insurance policies can influence anesthesiologists, and 68% showed faith in their anesthesiologist.

**Table 3:** Assessment of trust among physicians and anesthesiologists (n=406)

Assessment questions & answers	N (%)
Do you trust your doctor would prioritize your medical care and put all other considerations aside?	
Prefer not to answer	07 (01.7%)
Yes	259 (63.8%)
No	16 (03.9%)
Not sure	124 (30.5%)
Have you ever rejected receiving medical care because you lacked faith in your physician?	
Prefer not to answer	04 (01.0%)
Yes	91 (22.4%)
No	263 (64.8%)
Not sure	48 (11.8%)
Do you believe anesthesiologists are influenced by medical insurance rules while caring for you?	
Prefer not to answer	44 (10.8%)
Yes	39 (09.6%)
No	207 (51.0%)
Not sure	116 (28.6%)
Do you have faith that your anesthesiologist would set all other considerations aside and prioritize taking care of you?	
Prefer not to answer	07 (01.7%)
Yes	276 (68.0%)
No	21 (05.2%)
Not sure	102 (25.1%)

**Figure 1:**
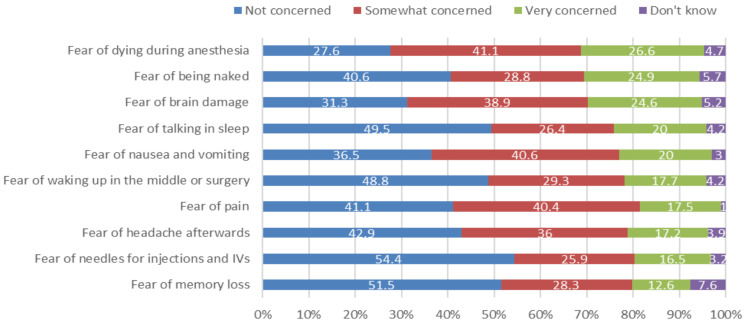
Concerns or fears about anesthesia

Figure 1 depicts the most common concerns about anesthesia, as rated by our respondents: "fear of dying during anesthesia" (very concerned: 26.6%), followed by "fear of being naked" (very concerned: 24.9%), "fear of brain damage" (very concerned: 20%), and "fear of nausea and vomiting" (very concerned: 20%).

Knowledge related to anesthesia

It can be observed that good knowledge related to anesthesia was seen in the statements, 'it is important for the anesthesiologist to know your medical history and your medication history before surgery' (true: 88.7%), followed by 'certain types of surgeries can be done by blocking nerves with local anesthetics without needing to be completely put to sleep' (true: 86%), 'every type of surgery requires patients to be put to sleep' (false: 84%), 'an anesthesiologist is a specially trained doctor' (true: 79.8%), and 'nausea and vomiting are frequent side effects of general anesthesia' (true: 74.9%). On the contrary, poor knowledge was seen in the statements, 'an anesthesiologist is an expert in the treatment of pain and takes care of pain after surgery' (true: 25.4%), 'there is an occasional chance of being aware of what’s going on under general anesthesia' (true: 31.3%), and 'fasting prior to surgery means you cannot have anything by mouth except water' (false: 32.3%). The overall mean knowledge score was 8.14 (SD 2.35). Poor, moderate, and good knowledge were detected in 20%, 67.7%, and 12.3%, respectively (Table 4).

**Table 4:** Assessment of knowledge related to anesthesia (n=406)

Statement	True N (%)	False N (%)	Not sure N (%)
An anesthesiologist is a specially trained doctor (TRUE)	324 (79.8%)	32 (07.9%)	50 (12.3%)
A specially trained nurse can be an anesthesia provider when supervised by an anesthesiologist (FALSE)	105 (25.9%)	165 (40.6%)	136 (33.5%)
An anesthesiologist is an expert in the treatment of pain and takes care of pain after surgery (TRUE)	103 (25.4%)	157 (38.7%)	146 (36.0%)
An anesthesiologist can give an epidural during childbirth (TRUE)	265 (65.3%)	38 (09.4%)	103 (25.4%)
Every type of surgery requires patients to be put to sleep (FALSE)	36 (08.9%)	341 (84.0%)	29 (07.1%)
Certain types of surgeries can be done by blocking nerves with local anesthetics without needing to be completely put to sleep (TRUE)	349 (86.0%)	40 (09.9%)	17 (04.2%)
It is important for the anesthesiologist to know your medical history and your medication history before surgery (TRUE)	360 (88.7%)	18 (04.4%)	28 (06.9%)
Fasting prior to surgery means absolutely nothing by mouth (TRUE)	247 (60.8%)	134 (33.0%)	25 (06.2%)
Fasting prior to surgery means you cannot have anything by mouth except water (FALSE)	232 (57.1%)	131 (32.3%)	43 (10.6%)
Overall, anesthesia is extremely safe (TRUE)	176 (43.3%)	128 (31.5%)	102 (25.1%)
General anesthesia frequently results in brain damage (FALSE)	87 (21.4%)	184 (45.3%)	135 (33.3%)
Overall risks of anesthesia are higher in sicker patients (TRUE])	228 (56.2%)	56 (13.8%)	122 (30.0%)
Nausea and vomiting are frequent side effects of general anesthesia (TRUE)	304 (74.9%)	32 (07.9%)	70 (17.2%)
There is an occasional chance of being aware of what’s going on under general anesthesia (TRUE)	127 (31.3%)	193 (47.5%)	86 (21.2%)
Total knowledge score (mean ± SD)	8.14 ± 2.35	--	--
Level of knowledge			
Poor	81 (20.0%)	--	--
Moderate	275 (67.7%)	--	--
Good	50 (12.3%)	--	--

**Figure 2:**
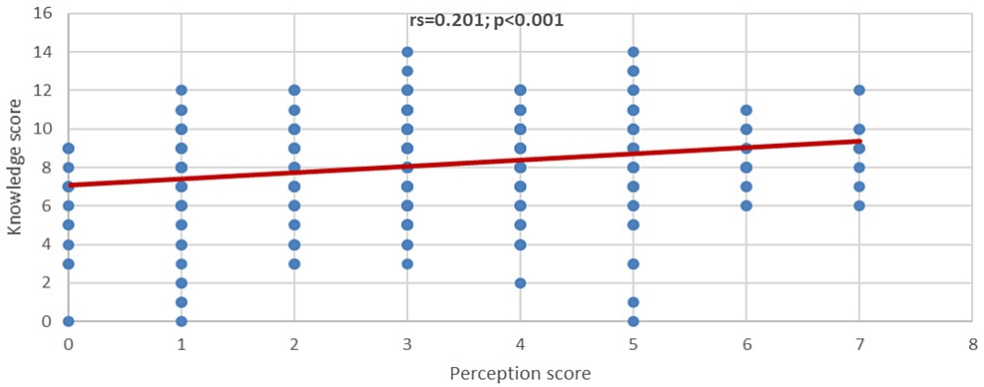
Correlation between perception score and knowledge score

It was observed that there was a significant positive correlation between perception score and knowledge score (rs=0.201; p<0.001) (Figure 2). Indicating that whenever the perception score increases, the knowledge score will also likely increase.

Perception and knowledge in relation to the Sociodemographic characteristics of the participants

When measuring the differences in the scores of perception and knowledge in relation to the sociodemographic characteristics of participants (Table 5), it was found that a higher perception score was associated more with having a chronic medical condition (Z=1.965; p=0.049), while a higher knowledge score was associated more with being a female (Z=1.446; p=0.020) and having a previous history of surgery (Z=2.136; p=0.033). No significant differences were observed between perception and knowledge scores in terms of age group, region of residence, or self-assessment of health status (p>0.05).

**Table 5:** Differences in the score of perception and knowledge in relation to the sociodemographic characteristics of the participants (n=406) § p-value has been calculated using Mann Whitney Z-test. ** Significant at p<0.05 level

Factors	Perception Score (7) Mean ± SD	Z-test; p-value ^§^	Knowledge Score (14) Mean ± SD	Z-test; p-value ^§^
Age group				
· <50 years	3.25 ± 1.51	0.115; 0.908	8.14 ± 2.46	0.468; 0.640
· ≥50 years	3.24 ± 1.69		8.13 ± 2.19	
Gender				
· Male	3.47 ± 1.39	1.047; 0.295	7.54 ± 3.03	1.446; 0.020 **
· Female	3.19 ± 1.62		8.26 ± 2.17	
Educational level				
· High school or below	3.12 ± 1.48	0.554; 0.580	7.84 ± 2.52	0.875; 0.381
· Bachelor or higher	3.26 ± 1.60		8.18 ± 2.33	
Region of residence				
· Inside Central Region	3.25 ± 1.63	0.112; 0.911	8.16 ± 2.34	0.207; 0.836
· Outside Central Region	3.23 ± 1.31		8.02 ± 2.43	
Chronic medical condition				
· Yes	3.53 ± 1.66	1.965; 0.049 **	7.95 ± 2.11	1.179; 0.238
· No	3.16 ± 1.56		8.19 ± 2.42	
Self-assessment of health status				
· Fair/Good	3.22 ± 1.71	0.142; 0.887	8.15 ± 2.47	0.080; 0.937
· Very good/Excellent	3.24 ± 1.61		8.33 ± 2.17	
Previous surgery				
· Yes	3.19 ± 1.61	1.034; 0.301	8.32 ± 2.28	2.136; 0.033 **
· No	3.35 ± 1.54		7.77 ± 2.46	

## Discussion

In the current study, many participants knew what an anesthesiologist does and how long it takes both anesthesiologists and physicians in general to attend medical school. However, not much was known about how long anesthesiologists spent in residency. Another study found that just 32% of participants felt fully informed about anesthesia. This might be due to a general lack of familiarity with the work of anesthesiologists and the mechanisms of anesthetics [12]. Another study had shown that patients in this group had high average education and health literacy, yet many still needed to understand what anesthesiologists do. Patients desire to learn as much as possible during the preoperative appointment. The most effective means of disseminating this data was via an informative pamphlet [12]. Most patients were aware that anesthesiologists administered anesthesia. Anesthesiologists were valued, but their role during surgery and the anesthetics they used were unknown. In another study, these findings suggest that anesthesiologists should educate surgical patients before surgery to establish rapport, distribute anesthesia education materials, and use the media to educate illiterate people about anesthesia [4].

No significant differences were observed in this study between perception and knowledge scores regarding age group, region of residence, and self-assessment of health status. A higher perception score was more associated with having a chronic medical condition (Z=1.965; p=0.049), while a higher knowledge score was more associated with being female (Z=1.446; p=0.020). Another similar study looked at how much people in the Qassim area of Saudi Arabia know about the field of anesthesia and the role of anesthesiologists. The results showed that individuals with a bachelor's degree or higher were more likely to have had surgery before. Anesthesia awareness was improved by knowing about regional anesthesia, but the sample showed that people didn't know much about anesthesia [13].

Table 3 shows that the majority (63.8%) of respondents trusted medical professionals. However, 22.4% declined medical care because they didn't trust physicians. Most of our respondents (51%) do not think that medical insurance laws may influence anesthesiologists, and 68% expressed confidence in their anesthesiologist. Another study examined anesthesiologists' roles, trust, knowledge, and anxieties among predominantly Hispanic patients from an inner-city county preoperative anesthesia clinic. This will discuss professionalism in anesthesiology, with an emphasis on empathy, social media usage, and awareness of drug use disorders, aiming to enhance the public image of anesthesiologists. Patient empathy can significantly enhance patient-provider trust [14]. In a similar study, it was found that 68% of patients trusted their doctors. The study also highlights the public's expectation for anesthesiologists to possess a certain level of clinical competence and technical knowledge to provide care for patients and maintain this competence throughout their careers [1].

Trust is the cornerstone of the relationship between patients and their physicians, and without it, the healthcare journey will be full of difficulties and obstacles. In our country, the culture of medical insurance wasn’t common among the Saudi population due to the governmental pledge to provide health care services for free to Saudi citizens. However, the recent trends of the Saudi government center around the privatization of governmental hospitals and reliance on health care insurance policies. The new strategy may influence the level of trust between Saudi patients and their anesthesiologists. In our survey, about 51% of the respondents were confident that their anesthesiologist’s decisions would not be influenced by insurance companies. This result represents a high level of trust when compared with another survey that was conducted among a predominantly Hispanic patient population in California, USA [1], where only 34.7% of their participants were sure about that.

Fear and anxiety about surgical procedures and anesthesia are common; some researchers found that 88.9% of preoperative patients showed an overall fear, whereas 10.8% of the surveyed participants were exclusively worried about anesthesia [15]. In this study, we noticed that the fear of dying during surgery was at the top of the list of our subjects' concerns toward anesthesia, as 26.6% of them addressed it as a significant concern. On the other hand, two prior studies [15,16] found that only 16.9% and 12.1% of their respondents showed the same level of fear. Nagrampa et al.'s results were similar to ours, where 29.3% of their patients were assessed to be "very concerned" about dying during surgery [1]. Interestingly, 24.9% of our participants chose nudity as a major fear, while in a Canadian study [16] only 3.6% of their respondents estimated it as a "very concerned" idea. This could be attributable to the nature of our sample, which is female-dominant, and the strong adherence to religious and cultural rules in our nation.

Trypanophobia and amnesia represent the least significant concerns among our surveyed sample. By contrast, another study was accomplished in India to assess the fears and perceptions associated with regional anesthesia [17], stating that trypanophobia was one of the top concerns among their patients. The reasons for this discrepancy are unclear, but the general public may underestimate certain aspects of medical procedures when compared with patients undergoing scheduled surgeries. 

Limitations

This study sample may not represent the general population or the world in which the research was conducted. Because of this, generalizing the findings to a wider population may be challenging. Subjects' self-reports served as the primary data source for this investigation. This allows individuals to express opinions based on what they believe others want to hear rather than what they know or think. The research used a cross-sectional approach, which only revealed participants' knowledge and attitudes during the survey. The evolution of knowledge and perspectives may be better understood through longitudinal research.

The research may have overlooked some additional factors that could have influenced individuals' attitudes or familiarity with anesthesia, as it appears to have focused primarily on a narrow set of variables related to Saudi citizens' perceptions of anesthesiologists' training, expertise, role, and responsibilities, as well as their knowledge and concerns about anesthesia.

Recommendations 

Future research may employ the following techniques to better fathom anesthesia and its practitioners: the participant pool should include a broader range of ages, ensuring equal representation of both genders, geographic locations, and socioeconomic backgrounds. This would enable a more comprehensive analysis and increase the applicability of the results. The design of a study that follows participants over time (a longitudinal study) could shed light on the actual efficacy of educational interventions or public awareness campaigns. Incorporating qualitative research methods, such as interviews or focus groups, could aid researchers in gaining a deeper understanding of the participants' perspectives on anesthesia. These anecdotes may round out the data and provide a wider picture. By developing new educational programs or public awareness campaigns, studies with an intervention seek to alter the way individuals view anesthesia and anesthesiologists. If the efficacy of these treatments were evaluated, it would be useful to have evidence-based methods for raising awareness.

## Conclusions

The findings suggest that while the majority of participants recognized anesthesiologists as specially trained doctors, there were still some misconceptions and knowledge gaps regarding their role and responsibilities. The study also highlights the importance of increasing public awareness about anesthesia and addressing common concerns, particularly the fear of dying during anesthesia. Healthcare providers should take steps to educate patients and address their concerns to improve patient satisfaction and trust. Overall, this study provides a foundation for future research on anesthesia perceptions and knowledge, which can inform efforts to enhance patient safety and satisfaction.

## Appendices

Appendix A

**Table 6:** Questionnaire assessing perceptions of anesthesiologist education, expertise, role, and responsibilities

Questions & statements about education, expertise, role, and responsibilities
1. Who puts you to sleep before surgery?
- Surgeon
- Anesthesiologist
- Nurse
- I don’t know
2. Who is responsible for waking you up after surgery?
- Surgeon
- Anesthesiologist
- Nurse
- I don’t know
3. Who is responsible for monitoring your vital signs throughout surgery?
- Surgeon
- Anesthesiologist
- Nurse
- I don’t know
Statement about education and training
4. The number of medical school years required for an anesthesiologist
- 4 or less
- 5 or more
- I don’t know
5. The number of medical school years required for surgeons
4 or less
5 or more
I don’t know
6. The number of residency training years required for an anesthesiologist
- 4 or less
- 5 or more
- I don’t know
7. The number of residency training years required for a surgeon
- 4 or less
- 5 or more
- I don’t know

Appendix B

**Table 7:** Questionnaire assessing trust in physicians and anesthesiologists

Questions	Yes	No	Not sure	Prefer not to answer
1. Do you trust your doctor would prioritize your medical care and put all other considerations aside?	◯	◯	◯	◯
2. Have you ever rejected receiving medical care because you lacked faith in your physician?	◯	◯	◯	◯
3. Do you believe that anesthesiologists are influenced by medical insurance rules while taking care of you?	◯	◯	◯	◯
4. Do you have faith that your anesthesiologist would set all other considerations aside and prioritize taking care of you?	◯	◯	◯	◯

Appendix C 

**Table 8:** Questionnaire assessing concerns or fears about anesthesia

Type of concern/fear	Very concerned	Somewhat concerned	Not concerned	Don’t know
Fear of pain	◯	◯	◯	◯
Fear of dying during anesthesia	◯	◯	◯	◯
Fear of brain damage	◯	◯	◯	◯
Fear of waking up in the middle of surgery	◯	◯	◯	◯
Fear of memory loss	◯	◯	◯	◯
Fear of headache afterwards	◯	◯	◯	◯
Fear of nausea and vomiting	◯	◯	◯	◯
Fear of needles for injections and IVs	◯	◯	◯	◯
Fear of being naked	◯	◯	◯	◯
Fear of talking in sleep	◯	◯	◯	◯

Appendix D 

**Table 9:** Questionnaire assessing knowledge related to anesthesia

Statements	True	False	Don't know
An anesthesiologist is a specially trained doctor.	o	o	o
A specially trained nurse can be an anesthesia provider when supervised by an anesthesiologist.	o	o	o
An anesthesiologist is an expert in the treatment of pain and takes care of pain after surgery.	o	o	o
An anesthesiologist can give an epidural during childbirth.	o	o	o
Every type of surgery requires patients to be put to sleep.	o	o	o
Certain types of surgeries can be done by blocking nerves with local anesthetics without needing to be completely put to sleep.	o	o	o
It is important for the anesthesiologist to know your medical history and your medication history before surgery.	o	o	o
Fasting prior to surgery means absolutely nothing by mouth.	o	o	o
Fasting prior to surgery means you cannot have anything by mouth except water.	o	o	o
Overall, anesthesia is extremely safe.	o	o	o
General anesthesia frequently results in brain damage.	o	o	o
Overall risks of anesthesia are higher in sicker patients.	o	o	o
Nausea and vomiting are frequent side effects of general anesthesia.	o	o	o
There is an occasional chance of being aware of what's going on under general anesthesia	o	o	o
